# Effectiveness of a grid mattress on adults' sleep quality and health: A quasi‐experimental intervention study

**DOI:** 10.1002/hsr2.2046

**Published:** 2024-04-18

**Authors:** Michael Breus, Stephanie L. Hooper, Tarah Lynch, Martin Barragan, Heather A. Hausenblas

**Affiliations:** ^1^ Independent Practice Hermosa Beach California USA; ^2^ Brooks College of Health University of North Florida Jacksonville Florida USA; ^3^ School of Applied Health Sciences, Brooks Rehabilitation College of Healthcare Sciences Jacksonville University Jacksonville Florida USA

**Keywords:** activity, mattress, mood, sleep quality

## Abstract

**Background and aims:**

Despite that 93% of people indicate that a mattress plays a pivotal role in achieving high‐quality sleep, there is a scarcity of research investigating the influence of mattresses on sleep quality, pain, and mood in nonclinical poor sleepers. The purpose was to examine the effectiveness of a pressure‐releasing medium‐firm grid mattress on sleep and health outcomes (e.g., mood, pain, daytime fatigue) of adults with nonclinical insomnia symptoms using a quasi‐experimental design.

**Methods:**

Participants were 39 adults (mean age = 45.29) with nonclinical insomnia (i.e., occasional sleeplessness). Following 1 week of baseline assessments on their current mattress, they slept on a pressure‐relieving grid mattress for 8 weeks. Participants completed self‐report assessments of the Pittsburgh Sleep Quality Index, Berlin Questionnaire, Insomnia Severity Index, Restorative Sleep Questionnaire, Perceived Stress Scale, Profile of Mood States, Daytime Fatigue Scale, Pain and Sleep Questionnaire, and Brief Pain Inventory at Baseline and Weeks 1, 2, 3, 4, and 8. Participants continually wore an Oura Ring to objectively assess sleep and daytime activity. The data were collected from January 2022 to April 2022 and were stored electronically. Repeated‐measures analyses of variance were used to analyze mean time differences.

**Results:**

Self‐reported sleep quality, perceived pain, perceived stress, mood, and daytime fatigue improved significantly from Baseline to Week 8, *p*'s < 0.05. Objective Oura Ring validated the self‐reported sleep and daytime activity outcomes with improvements in sleep duration, time awake during the night, light sleep, deep sleep, and total sleep time, *p*'s < 0.05. No significant time effects were evidenced for rapid eye movement sleep. No adverse events were reported.

**Conclusion:**

The grid mattress is a simple, noninvasive, and nonpharmacological intervention that improved adults sleep quality and health. Controlled trials are encouraged to examine the effects of this mattress in a variety of populations and environments.

## Key points

**What's Known:**

- Although numerous studies recognize that mattresses are an important environmental factor on sleep quality, limited research exists on which mattress design is optimum for improving sleep quality and overall health, especially in the natural home environment.
- Medium‐firm mattresses provide the best sleep quality but their effects on nonclinical poor sleepers requires more research.

**What's New:**

- A quasi‐experiment intervention involving 39 adults with poor sleep quality examined the impact of a pressure‐relieving medium‐firm grid mattress on sleep quality and health.
- Sleeping on the grid mattress led to significantly improved sleep quality, increased daytime activity, reduced daytime fatigue, improved mood, and lowered anxiety/stress.
- Clinical implications.
- The grid mattress may be a nonpharmacological approach for promoting sleep quality, daytime activity, and overall health in people with occasional sleeplessness.

## 1 INTRODUCTION

Sleep is essential to a variety of health domains, including weight management, mood regulation, longevity, positive social interactions, academic/occupational/sport performance, and overall quality of life.^1^, ^2^ Humans should sleep for about a third of their lifetime, which equates to 7–9 h a night. Despite its health importance, most adults get less than the recommended hours of nighttime sleep, with almost 80% of adults reporting poor sleep quality.^3^

The most common interventions to improve sleep quality are over‐the‐counter and prescription drugs, which often have side effects, limited efficacy, and may result in dependency.^4^ Thus, there is an urgent need to examine the effectiveness of nonpharmacological interventions of lifestyle choices and environmental conditions to improve sleep and related health outcomes.^5^

Although numerous studies recognize that mattresses are an important environmental factor for sleep quality and health,^6^, ^7^, ^8^ limited research exists on which mattress design is optimum for improving sleep quality, perceptions of pain, and overall health,^9^ especially in the natural home environment (as opposed to settings such as hospitals and labs) with nonclinical populations using validated objective and self‐report measures.

The National Sleep Foundation, USA, has emphasized the significance of a comfortable mattress, with 93% of people recognizing it as a critical factor for achieving high‐quality sleep.^10^ Mattresses are important environmental components of sleep quality, consequently exerting an influence on overall health.^11^, ^12^

The negative consequences of poor sleep quality are severe enough to research which is the best mattress to promote quality sleep. Previous studies reported that the mechanical characteristics of the mattress can play a key role for sleep quality, with two systematic reviews finding that medium‐firm mattresses promote comfort, sleep quality, and spinal alignment. In addition, medium‐firm mattresses are perceived as more comfortable than soft bedding systems.^6^, ^9^

In short, despite the importance, ecologically valid research is needed examining the effectiveness of mattresses in the natural environment on sleep quality and related health outcomes in adults with poor sleep quality with both self‐report and objective assessments. Thus, the purpose of this study was to examine the effectiveness of a pressure‐releasing medium‐firm grid mattress on sleep and health outcomes in adults with nonclinical insomnia in their natural home environment using both self‐report and validated wearable assessments. The primary outcomes were sleep quality/quantity. The secondary outcomes were perceived stress, pain, daytime productivity, mood, daytime fatigue, and adverse events. It was hypothesized that sleeping on the grid mattress would result in improved sleep and health outcomes compared to baseline.

## 2 METHODS

### 2.1 Participants

**Figure 1:**
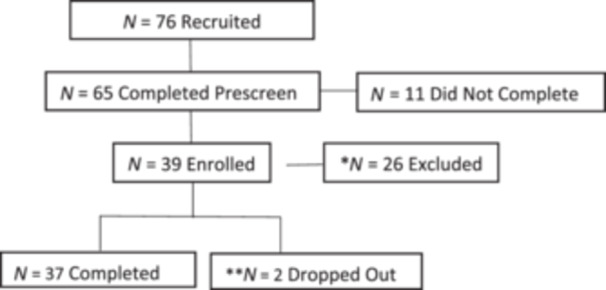
Participant flow chart. *N = 26 excluded, because they did not meet the inclusion criteria (*N* = 3 body mass index [BMI] too high, *N* = 10 insomnia scores were too low, *N* = 2 smokers, *N* = 4 enrollment was full, and *N* = 7 stopped responding after prescreen). ** *N* = 2 dropped out due medical condition unrelated to the study.

Participants were 39 male (*n *= 12) and female (*n* = 27) adults (mean [M] age = 45.29, SD = 6.12, range = 32–57 years) with a body mass index (BMI) of <35 (M BMI = 33.72, SD = 19.80), who reported poor sleep quality (as determined by the Insomnia Severity Index [ISI];^13^ M ISI = 14.54, SD = 3.66, range = 8–22) and low risk for sleep apnea (as determined by the Berlin Questionnaire); see Figure 1.

### 2.2 Exclusion criteria

Participants were excluded if they had severe insomnia (based on the ISI [score ≥ 22] or absence of insomnia [score < 8]); (2) history of a disorder affecting sleep quality; (3) events that could cause severe stress within 4 weeks of baseline; (4) medications that could influence sleep patterns, within 1 month of the study; (5) hormone therapy; (6) binge drinking—classified as >140 g/week, 2.5 bottles/week of alcohol, 2.5 shots/day; (7) smoking; (8) high caffeine intake defined as more than 400 mg of caffeine/day; (9) work schedule that causes irregular sleep patterns; (10) travel to a different time zone within 1 month of study; (11) low or high BMI (≤18 or ≥35 kg/m^2^); (12) currently pregnant, trying to conceive, or breastfeeding; (13) taking sleep supplements or medication, (14) high risk for sleep apnea according to the Berlin Questionnaire criteria of high risk of obstructive sleep apnea, and (15) individuals deemed incompatible with the protocol.

### 2.3 Intervention

The Purple Grid™ mattress is a pressure‐relieving medium‐firm mattress that is made of polyurethane‐foam, better mineral oil, and durable thermoplastic rubber. The GelFlex^(R)^ hyper‐elastic polymer grid is a repeating geometric structure that is engineered to instantly adapt to movement, reconfiguring to support any body position. The grid consists of 1400 columns that provides support and pressure relief by adapting to body contours, and it is purported to reduce discomfort/pain and enhance sleep quality. As weight is applied to the mattress the grid buckles, which is purported to result in pressure relief. The mattress also incorporates foam layers for durability and breathability for temperature regulation. The grid material is 100% hypoallergenic made with recyclable food grade and food‐contact grad material. All material in the mattress is clean air GOLD and Certi‐PUR‐US certified.

### 2.4 Study design

The independent variable was the grid mattress. The dependent variables were sleep quality, mood, perceived pain, perceived stress, and anxiety. Sample size power calculation indicated that 38 participants were needed to achieve a power of 80% and *α* < 0.05 (https://clincalc.com/stats/samplesize.aspx). Following 1 week of baseline assessments on the participants' current mattress, they slept on a grid mattress for 8 weeks in their natural home environment.

### 2.5 Procedures

This study was reviewed by a research ethics board of WCG IRB (Protocol Number: 1317666, Approval Date: 09/20/2021). This study was conducted in accordance with the ethical principles that originate in the Declaration of Helsinki and its subsequent amendments. Informed consent was obtained from each participant before performing any study‐related activities.

Interested participants completed a Prescreen Survey to determine eligibility. All the participants started and ended the study at the same time to control for seasonal and holiday effects on sleep. As a decentralized trial, participants did not visit a clinic, all recruitments, contact, screening, consenting and assessments were performed online.

Participants slept on their current mattress for 1 week (Baseline Assessment). Then the participants slept on the grid mattress for 8 weeks. Each day the participants completed a Daily Diary that assessed adverse events and adherence. Also, the participants completed assessments of their sleep quality, pain, daytime fatigue, and perceived stress at Baseline, and Weeks 1, 2, 3, 4, and 8. Participants wore the Oura Ring 24/7 as an objective measure of sleep quality and daytime activity (except when charging).

To reduce external, contraindicating factors, the participants slept in their own bedrooms, with their personal linens and pillows, and self‐selected thermal environment. Participants maintained their lifestyle behaviors and did not start a new exercise, diet, or health intervention during this study. In addition, participants maintained a daily diary to document adherence and adverse events. Participants completed the self‐report surveys via a SurveyMonkey link that was sent via email or text. Completion of the surveys took about 25 min at each assessment. Participants were instructed to maintain their habitual lifestyle patterns and refrain from introducing new exercise, diet, or health interventions during the study. Data were collected from March 2022 to April 2022 and were stored electronically.

### 2.6 Adverse events

The mattress was well‐tolerated and no adverse events were reported.

### 2.7 Trial reporting

The Consolidated Standards of Reporting Trials (including reporting of harms) was used to report this trial.

### 2.8 Adherence

Thirty‐nine participants enrolled and consented, of whom 37 completed the intervention, representing an adherence rate of 95%. The two participants dropped out due to personal reasons unrelated to the study (see Figure 1).

### 2.9 Statistical analysis

Data were analyzed for normality using Shapiro–Wilk test, visual inspection of *Q*–*Q* plots, and the skewness and kurtosis values of the scales, and we found that the distribution were normal. Descriptive statistics were expressed in M and SD scores. Repeated‐measures analysis of variance (ANOVA) were used to analyze the main outcomes across times (*p*'s ≤ 0.05) with the effect size of partial *η* ^2^. Posthoc tests (e.g., pairwise comparisons) with Bonferroni correction were undertaken when the ANOVA was significant. Moderator analysis of gender, age, and sleep quality were examined via one‐way ANOVAs. All data analyses were conducted using Statistical Package for the Social Sciences version 24 statistical software from IBM company, with an a priori *α*‐level of 0.05. We used two‐sided tests for all variables.

## 3 MEASURES

### 3.1 Insomnia Severity Index (ISI)

The ISI is a seven‐item self‐report measure assessing insomnia symptoms. This index assesses sleep onset, sleep maintenance, early morning awakening problems; sleep dissatisfaction; interference of sleep difficulties with daytime functioning; whether sleep problems are noticed by others; and distress caused by sleep difficulties. A 5‐point Likert scale is used to rate each item (e.g., 0 = *no problem*; 4 = *very severe problem*), yielding a total score ranging from 0 to 28. The total score is interpreted as follows: absence of insomnia (0–7); subthreshold insomnia (8–14); moderate insomnia symptoms (15–21); and severe insomnia symptoms (22–28). The ISI has excellent internal consistency (Cronbach *α* = 0.91).^13^

### 3.2 Pittsburgh Sleep Quality Index

The Pittsburgh Sleep Quality Index is a well‐known, validated, and reliable instrument used to measure sleep quality. This Index assesses the following seven components: (1) perceived sleep quality; (2) sleep latency (how long it takes to fall asleep); (3) sleep duration; (4) habitual sleep efficiency (how long a person is asleep in comparison to their time in bed); (5) sleep disturbances (i.e., noise, temperature, pain, nocturia); (6) sleep medications; and (7) daytime dysfunction (sleepiness, concentration). This inventory has good overall reliability and validity. A score > 5 indicates impaired sleep quality.^14^

### 3.3 Berlin Questionnaire

The Berlin Questionnaire assesses the symptoms for the diagnosis of obstructive sleep apnea. This questionnaire comprises the following three categories: (1) snoring and apnea; (2) daytime sleepiness or fatigue, and (3) information about obesity and hypertension. High risk of obstructive sleep apnea is defined as ≥2 positive results of the three categories.^15^

### 3.4 Restorative Sleep Questionnaire

The Restorative Sleep Questionnaire measures refreshing quality of sleep. Nonrestorative sleep is one of the cardinal symptoms of insomnia and can occur independent of other components of insomnia. This questionnaire has nine items with answers scaled from 1 to 5. The total score is an average score based on all nine items. This questionnaire has good psychometric properties.^16^

### 3.5 Profile of Mood States (POMS) Questionnaire

The POMS‐40 was used to assess the mood states of tension, anger, vigor, fatigue, depression, and confusion. A composite score was computed by summing each of the individual scores for tension, depression, anxiety, fatigue, and confusion, with vigor scores subtracted to indicate patients' total mood disturbance. Each item of the POMS items was scored on a 5‐point Likert scale ranging from 0 (*not at all*) to 4 (*extremely*) with lower scores indicated an improved mood. This scale has good to excellent reliability and validity.^17^

### 3.6 Flinders Fatigue Scale

The Flinders Fatigue Scale is a seven‐item scale that measures various characteristics of daytime fatigue (e.g., frequency, severity) experienced over the past 2 weeks. The items tap into commonly reported themes of how problematic fatigue is, the consequences of daytime fatigue, and perception of fatigue's association with sleep. Six items are presented in Likert format, with responses ranging from 0 (*not at all*) to 4 (*extremely*). Item 5 measures the time of day when fatigue is experienced and uses a multiple‐item checklist. Respondents can indicate more than one response for item 5 and it is scored as the sum of all times of the day indicated by the respondent. One item explicitly asks for respondents' impression of whether they attribute their fatigue to their sleep. Total fatigue is calculated as the sum of all individual items (range = 0–31), with higher scores indicating greater fatigue. A description of the term “fatigue” is provided in the initial instructions to the scale of: “We are interested in the extent that you have felt fatigued (tired, weary, exhausted). We do not mean feelings of sleepiness (the likelihood of falling asleep)”.^18^

### 3.7 Perceived Stress Scale

The Perceived Stress Scale‐4 measures the degree to which individuals appraise situations in their lives as stressful.^19^ Specifically, the scale evaluates the degree to which individuals believe their life has been unpredictable, uncontrollable, and overloaded during the previous month. The items are general in nature rather than focusing on specific events or experiences. The scale has four items, and each item was scored on a 5‐point Likert scale ranging from 0 (*never*) to 4 (*very often*) with higher scores indicating more perceived stress. This scale has excellent psychometric properties.^19^, ^20^

### 3.8 Pain and Sleep Questionnaire Three‐item Index

The Pain and Sleep Questionnaire Three‐Item Index is a brief, simple, and psychometrically sound measure of the impact of chronic pain on sleep. The following three items are assessed on a scale ranging from 0 (*never*) to 100 (*always*): (1) How often do you have trouble falling asleep?; (2) How often are you awakened by pain during the night?; and (3) How often are you awakened by pain in the morning? This index has strong reliability and structural, criterion‐related, and predictive validity.^21^

### 3.9 Brief Pain Inventory

The Brief Pain Inventory is a nine‐item self‐administered questionnaire used to evaluate the severity of a person's pain and the impact of this pain on the person's daily functioning. Participants rate their worst, least, average, and current pain intensity, list current treatments and their perceived effectiveness, and rate the degree that pain interferes with general activity, mood, walking ability, normal work, relations with other persons, sleep, and enjoyment of life on a 10‐point scale. The brevity of the Brief Pain Inventory makes it suitable for settings in which pain is assessed frequently (e.g., intervention).^20^

### 3.10 Daily diary

The daily diary assessed adverse events and adherence.

### 3.11 Oura Ring

The Oura Ring is an objective multisensory wearable device that quantifies nighttime sleep duration and estimates sleep stages, including rapid eye movement (REM; https://ouraring.com/). The Oura Ring uses physiological signals (a combination of motion, heart rate, heart rate variability, and pulse wave variability amplitude) in combination with sophisticated machine learning‐based methods to calculate deep, light, and REM sleep in addition to sleep/wake states. Rings are waterproof, made in ceramic, and come with a dedicated mobile App. The ring automatically connects via Bluetooth and transfers data to a mobile platform via the dedicated App. The Oura Ring has high validity in the assessment of nocturnal heart rate, heart rate variability, movement, and sleep outcomes in healthy adults in natural environment.^22^, ^23^

## 4 RESULTS

### 4.1 Primary outcomes: Sleep quality and quantity

For sleep quality significant improvements from Baseline to Week 8 were evidenced for the Pittsburgh Sleep Quality Index, Berlin Questionnaire, ISI, and Restorative Sleep Questionnaire, *p*'s < 0.05 (see Table 1).

**Table 1:** Self‐reported sleep quality/quantity Mean (M), Standard Deviation (SD), and repeated‐measures analyses of variance (F) Scores.

	Baseline M (SD)	Week 1 M (SD)	Week 2 M (SD)	Week 3 M (SD)	Week 4 M (SD)	Week 8 M (SD)	ANOVA F (5)	Partial *η* ^2^ omnibus	Partial *η* ^2^ posthocs
Pittsburg Sleep Quality Index^a^	7.49 (2.88)	5.46 (2.85)	5.54 (2.78)	5.61 (2.84)	5.14 (2.73)	4.92 (2.63)	*F* = 9.35, *p* ≤ 0.001	0.206	0.550
Restorative Sleep Questionnaire^a^	49.77 (16.66)	59.16 (20.40)	60.96 (19.50)	56.17 (18.25)	63.06 (19.46)	64.71 (18.49)	*F* = 7.15, *p* < 0.001	0.270	0.518
Berlin Questionnaire^a^	1.68 (1.75)	1.14 (1.51)	1.11 (1.54)	1.00 (1.39)	1.08 (1.38)	1.05 (1.41)	*F* = 13.33, *p* < 0.001	0.166	0.555
ISI^a^	12.11 (4.98)	9.08 (4.00)	8.30 (4.33)	8.69 (4.14)	7.22 (3.66)	7.05 (4.22)	*F* = 18.28, *p* < 0.001	0.337	0.588
Time to fall asleep (minutes)	16.76 (19.84)	13.80 (14.04)	13.42 (11.52)	14.91 (14.05)	15.88 (14.91)	13.42 (14.82)	*F* = 1.72, *p* = 0.127	0.009	0.040
Number of night awakenings^a^	2.60 (2.19)	2.01 (1.71)	2.18 (2.21)	2.41 (2.44)	1.91 (1.81)	2.05 (2.08)	*F* = 3.05, *p* = 0.009	0.016	0.080
Time awake during the night (minutes)^a^	49.76 (64.58)	34.18 (46.35)	37.17 (52.69)	38.31 (45.73)	30.51 (36.36)	30.75 (37.28)	*F* = 4.27, *p* < 0.001	0.022	0.084
Total sleep time Daily Diary	7.63 (1.68)	7.64 (1.67)	7.92 (2.25)	8.36 (1.82)	8.08 (2.48)	8.10 (2.05)	*F* = 3.05, *p* = 0.010	0.016	0.095

*Note*: Higher scores for Restorative Sleep Questionnaire and Total Sleep Time indicate improvements. Lower scores for Pittsburgh Sleep Quality Index, ISI, Time to fall asleep, Number of night awakenings, and Time awake during the night indicate improvements.

Pittsburg Sleep Quality Index: Significant differences were noted for Baseline to all Weeks (*p* < 0.001).

Berlin Questionnaire: Significant differences were noted for Baseline to Weeks 1–4 and 8 (*p* < 0.001), Week 1 to Week 4 (*p* < 0.001), Week 2 to Week 4 (*p* < 0.001), Week 3 to Week 4 (*p* < 0.001), and Week 4 to Week 8 (*p* < 0.001).

Restorative Sleep Questionnaire: Significant differences were noted for Baseline to Weeks 1–4 and 8 (*p* < 0.001), Week 3 to Week 4 (*p* = 0.004), and Week 3 to Week 8 (*p* < 0.001).

Insomnia: Significant differences were noted for Baseline to Weeks 1–4 and 8 (*p* < 0.001), Week 1 to Week 4 (*p* = 0.001), Week 3 to Week 4 (*p* = 0.037), Week 1 to Week 8 (*p* < 0.001), Week 2 to Week 4 (*p* = 0.37), Week 2 to Week 8 (*p* = 0.016), Week 3 to Week 4 (*p* = 0.016), and Week 3 to Week 8 (*p* = 0.008).

Time to fall asleep: Significant differences were noted for Baseline to Week 2 (*p* = 0.045).

Number of night awakenings: Significant differences were noted for Baseline to Week 1 (*p* = 0.006), Baseline to Week 4 (*p* < 0.001), Baseline to Week 8 (*p* = 0.004), and Week 3 to Week 4 (*p* = 0.035).

Time awake during night: Significant differences were noted for Baseline to Week 1 (*p* = 0.016), Baseline to Week 2 (*p* = 0.038), Baseline to Week 3 (*p* = 0.042), Baseline to Week 5 (*p* < 0.001), Baseline to Week 8 (*p* < 0.001), and Week 3 to Week 8 (*p* = 0.045).

Total sleep time: Significant differences were noted for Baseline to Week 8 (*p* = 0.041), Week 1 to Week 3 (*p* < 0.001), and Week 2 to Week 3 (*p* = 0.036).

Abbreviations: ANOVA, analysis of variance; ISI, Insomnia Severity Index; M, mean.

^a^ Significant improvements from Baseline to Weeks 1–4 and 8, *p*'s < 0.05.

Specifically, ISI and Global PSQI scores continually improved from Baseline to Weeks 1 (*p* < 0.001), 2 (*p* < 0.001), 3 (*p* < 0.001), 4 (*p* < 0.001), and 8 (*p* < 0.001). Restorative sleep, based on the Restorative Sleep Questionnaire, significantly improved from Baseline to Week 1 (*p* = 0.002), 2 (*p* < 0.001), 3 (*p* = 0.034), 4 (*p* < 0.001), and 8 (*p* < 0.001). Sleep apnea symptoms, based on the Berlin Questionnaire, improved significantly from Baseline to Weeks 1 (*p* = 0.027), 2 (*p* = 0.21), 3 (*p* = 0.14), 4 (*p* < 0.001), and 8 (*p* = 0.021). Gender differences were evidenced for the ISI, with larger improvements in insomnia symptoms evidenced for the women compared to the men, *p* = 0.023.

The Oura Ring data confirmed the self‐reported improvements in sleep duration, time awake during the night, light sleep, deep sleep, and total sleep time, *p*'s < 0.05 (see Table 2). No significant time differences were evidenced for REM sleep.

**Table 2:** Objective Sleep Scores (Oura Ring), Mean (M), Standard Deviation (SD) scores in minutes, and repeated‐measures ANOVA F statistics.

	Baseline M (SD)	Week 1 M (SD)	Week 2 M (SD)	Week 3 M (SD)	Week 4 M (SD)	Week 8 M (SD)	ANOVA F (5)	Partial *η* ^2^ omnibus	Partial *η* ^2^ posthocs
Sleep duration	482.13 (70.00)	483.63 (69.00)	492.93 (72.18)	506.12 (79.60)	497.74 (65.78)	488.86 (68.47)	*F* = 4.16, *p* < 0.001	0.018	0.083
Time awake	62.54 (32.84)	62.87 (32.00)	64.99 (31.61)	74.25 (40.3)	70.62 (36.9)	63.96 (30.53)	*F* = 4.59, *p* < 0.001	0.020	0.078
Light sleep	240.56 (52.99)	240.36 (47.21)	248.52 (57.7)	257.83 (56.39)	249.64 (54.49)	244.15 (52.47)	*F* = 4.76, *p* < 0.001	0.021	0.097
REM sleep	93.4 (31.81)	87.34 (30.42)	91.55 (29.45)	93.10 (29.76)	91.77 (29.41)	92.94 (31.8)	*F* = 1.38, *p* = 0.246	0.006	0.029
Deep sleep	77.43 (30.58)	81.12 (31.84)	79.39 (30.07)	74.18 (34.53)	76.61 (33.28)	80.33 (29.21)	*F* = 2.12, *p* = 0.061	0.009	0.042
Total sleep	417.14 (62.79)	414.41 (60.20)	421.18 (65.71)	432.45 (65.23)	423.86 (55.08)	420.29 (61.09)	*F* = 2.89, *p* = 0.013	0.013	0.065

*Note*: Total sleep = light + REM + deep sleep in minutes. Sleep duration = time awake + light sleep + REM sleep + deep sleep + sleep latency in minutes.

Sleep duration: Significant differences were noted for Baseline Week 3 (*p* < 0.001), Week 1 to Week 3 (*p* < 0.001), and Week 1 to Week 4 (*p* = 0.006).

Time awake: Significant differences were noted for Baseline to Week 3 (*p* = 0.002), Baseline to Week 4 (*p* = 0.018), Week 1 to Week 3 (*p* < 0.001), Week 1 to Week 4 (*p* = 0.008), Week 2 to Week 3 (*p* = 0.002), and Week 3 to Week 8 (*p* = 0.003).

Light sleep: Significant differences were noted for Baseline to Week 3 (*p* < 0.001), Week 1 to Week 2 (*p* = 0.035), Week 1 to Week 3 (*p* < 0.001), and Week 3 to Week 8 (*p* = 0.004).

REM sleep: Significant differences were noted for Baseline to Week 1 (*p* = 0.054), and Week 1 to Week 3 (*p* = 0.018).

Deep sleep: Significant differences were noted for Week 1 to Week 3 (*p* = 0.014), Week 1 to Week 4 (*p* = 0.036), and Week 3 to Week 8 (*p* = 0.014).

Total sleep: Significant differences were noted for Week 1 to Week 3 (*p* < 0.001).

Abbreviations: ANOVA, analysis of variance; M, mean; REM, rapid eye movement.

### 4.2 Secondary outcomes: Mood, pain, stress, and daytime fatigue

Significant improvements in the POMS (i.e., tension, anger, vigor, fatigue, confusion, and total mood), perceived stress, daytime fatigue, and nighttime and daytime pain were evidenced from Baseline to Week 8, *p*'s < 0.05 (see Table 3).

**Table 3:** Perceived stress, POMS, Flinder's Fatigue Scale, Perceived Stress Scale, and Pain Outcomes M, SD scores, and repeated‐measures analyses of variable (F) (ANOVA) statistics.

	Baseline M (SD)	Week 1 M (SD)	Week 2 M (SD)	Week 3 M (SD)	Week 4 M (SD)	Week 8 M (SD)	ANOVA F (5)	Partial *η* ^2^ omnibus	Partial *η* ^2^ posthocs
POMS Tension	7.35 (4.61)	5.81 (4.33)	5.08 (4.23)	4.89 (3.70)	3.70 (3.70)	4.03 (3.32)	*F* = 15.42, *p* < 0.001	0.300	0.530
POMS Anger	4.89 (4.12)	3.51 (3.83)	2.97 (2.95)	3.03 (2.64)	2.38 (2.94)	2.57 (2.52)	*F* = 7.18, *p* < 0.001	0.166	0.317
POMS Fatigue	8.46 (4.32)	6.86 (4.04)	6.27 (4.31)	6.06 (3.81)	4.86 (3.93)	4.59 (3.56)	*F* = 15.15, *p* < 0.001	0.296	0.628
POMS Depression	2.76 (3.30)	2.51 (3.01)	2.05 (2.36)	1.97 (2.22)	1.65 (2.12)	1.78 (2.79)	*F* = 1.67, *p* = 0.145	0.044	0.117
POMS Esteem	16.65 (3.11)	17.03 (3.30)	17.49 (3.23)	17.06 (3.61)	17.92 (3.29)	17.76 (2.99)	*F* = 2.29, *p* = 0.048	0.060	0.282
POMS Vigor	8.97 (4.40)	9.84 (5.01)	10.46 (4.73)	9.78 (4.25)	10.00 (4.45)	10.43 (4.22)	*F* = 1.76, *p* = 0.123	0.047	0.200
POMS Confusion	3.89 (2.63)	3.59 (2.94)	2.62 (2.20)	2.64 (2.38)	2.51 (2.73)	2.68 (2.86)	*F* = 7.67, *p* < 0.001	0.176	0.462
POMS‐Total	152.97 (14.67)	149.16 (12.52)	146.95 (11.34)	145.42 (10.23)	143.03 (10.94)	143.84 (9.91)	*F* = 11.84, *p* < 0.001	0.248	0.404
Flinder's Fatigue Scale	17.43 (5.47)	14.86 (4.91)	15.19 (5.35)	15.72 (4.69)	13.65 (4.6)	12.84 (4.8)	*F* = 7.07, *p* < 0.001	0.164	0.383
Perceived Stress Scale	4.68 (2.86)	4.19 (2.70)	3.92 (2.73)	3.92 (2.79)	3.49 (2.66)	3.70 (2.84)	*F* = 2.20, *p* = 0.05	0.057	0.219
Pain and Sleep Questionnaire Three‐item Index	6.22 (6.21)	4.76 (4.05)	5.03 (3.86)	5.42 (4.4)	4.35 (3.3)	4.43 (3.41)	*F* = 2.29, *p* = 0.077	0.053	0.125
Brief Pain Inventory: Pain Severity	1.83 (1.94)	1.01 (1.47)	1.09 (1.32)	1.17 (1.57)	0.73 (1.18)	0.74 (1.13)	*F* = 5.57, *p* < 0.001	0.134	0.361
Brief Pain Inventory: Pain Interference	0.93 (1.18)	0.57 (0.90)	0.62 (0.93)	0.80 (1.22)	0.48 (0.83)	0.65 (1.12)	*F* = 1.25, *p* = 0.287	0.034	0.208

*Note*: Higher scores for POMS Esteem and Vigor indicate an improvement. Lower scores for POMS Tension, Fatigue, Depression, Confusion, and Total, Flinder's Fatigue Scale, Perceived Stress Scale, Pain and Sleep Questionnaire, and Brief Pain Inventory Items (Severity and Pain Interference) indicate an improvement.

POMS Tension: Significant differences were noted for Baseline to Weeks 1–4 and 8 (*p* < 0.001), Week 1 to Week 3 (*p* = 0.024), Week 1 to Week 4 (*p* < 0.001), Week 1 to Week 8 (*p* < 0.001), Week 2 to Week 4 (*p* = 0.006), Week 2 to Week 8 (*p* = 0.039), and Week 3 to Week 4 (*p* = 0.006).

POMS Anger: Significant differences were noted for Baseline to Week 1 (*p* = 0.004) Baseline to Week 2 (*p* < 0.001), Baseline to Week 3 (*p* = 0.003), Baseline to Week 4 (*p* < 0.001), Baseline to Week 8 (*p* < 0.001), Week 1 to Week 4 (*p* = 0.047), and Week 1 to Week 8 (*p* = 0.040).

POMS Fatigue: Significant differences were noted for Baseline to Weeks 1 (*p* = 0.004), Baseline to Weeks 2, 3, 4, and 8 (*p* < 0.001), Week 1 to Week 4 (*p* < 0.001), Week 1 to Week 8 (*p* < 0.001), Week 2 to Week 4 (*p* = 0.028), Week 2 to Week 8 (*p* = 0.002), Week 3 to Week 4 (*p* = 0.028), and Week 3 to Week 8 (*p* = 0.001).

POMS Depression: Significant differences were noted for Baseline to Week 4 (*p* = 0.049).

POMS Esteem: Significant differences were noted for Baseline to Week 4 (*p* = 0.010), Week 1 to Week 4 (*p* = 0.045), and Week 3 to Week 4 (*p* = 0.038).

POMS Vigor: Significant differences were noted for Baseline to Week 2 (*p* = 0.015) and Baseline to Week 8 (*p* = 0.044).

POMS Confusion: Significant differences were noted for Baseline to Weeks 2–4 (*p* < 0.001), Baseline to Week 8 (*p* = 0.002), for Week 1 to Week 2 (*p* = 0.002), Week 1 to Week 3 (*p* = 0.001), Week 1 to Week 4 (*p* = 0.002), and Week 1 to Week 8 (*p* = 0.008).

POMS‐Total: Significant differences were noted for Baseline to Weeks 1 (*p* = 0.011), Baseline to Weeks 2–4 and 8 (*p* < 0.001), Week 1 to Week 3 (*p* = 0.016), Week 1 to Week 4 (*p* < 0.001), Week 1 to Week 8 (*p* < 0.001), Week 2 to Week 4 (*p* = 0.004), Week 2 to Week 8 (*p* = 0.012), and Week 3 to Week 4 (*p* = 0.041).

Flinder's Fatigue Scale: Significant differences were noted for Baseline to Week 1 (*p* = 0.005), Baseline to Week 2 (*p* = 0.044), Baseline to Week 4 (*p* < 0.001), Baseline to Week 8 (*p* < 0.001), Week 1 to Week 8 (*p* = 0.006), Week 2 to Week 8 (*p* = 0.013), Week 3 to Week 4 (*p* = 0.009), and Week 3 to Week 8 (*p* < 0.001).

Perceived Stress Scale: Significant differences were noted for Baseline to Week 4 (*p* = 0.004) and Baseline to Week 8 (*p* = 0.032).

Pain and Sleep Questionnaire: Significant differences were noted for Baseline to Week 4 (*p* = 0.044).

Brief Pain Inventory: Pain Severity: Significant differences were noted for Baseline to Week 1 (*p* = 0.001), Baseline to Week 2 (*p* = 0.022), Baseline to Week 3 (*p* = 0.011), Baseline to Week 4 (*p* < 0.001), and Baseline to Week 8 (*p* < 0.001).

Brief Pain Inventory: Pain Interference: Significant differences were noted for Baseline to Week 1 (*p* = 0.027) and Baseline to Week 4 (*p* = 0.05).

Abbreviations: ANOVA, analysis of variance; M, means; POMS, Profile of Mood States.

Specifically, tension, anger, fatigue, depression, and total mood (based on the POMS) improved significantly from Baseline to all weeks (i.e. Weeks 1–4 and 8). Esteem showed a significant improvement from Baseline to Week 4, Vigor from Baseline to Weeks 2 and 8, and Confusion from Baseline to Weeks 2–4 and 8.

For daytime fatigue, the Flinder's Fatigue Scale showed significant improvements from Baseline to all weeks (i.e., Weeks 1–4 and 8). Regarding stress, the Perceived Stress Scale scores improved significantly from Baseline to Weeks 4 and 8. Furthermore, for pain, the Pain and Sleep Questionnaire showed significant improvements from Baseline to Week 4 and the Brief Pain Inventory evidenced significant improvements for pain severity from Baseline to all weeks (i.e., Weeks 1–4 and 8), and for pain interference from Baseline to Weeks 1 and 4.

No adverse events were reported. Moderator analysis by gender, age, and sleep quality (ISI) revealed no significant effects, *p*'s > 0.05.

## 5 DISCUSSION

Lack of sleep negatively impacts a person's cognitive and physical performances, mood, quality of life, social interaction, and can lead to increased daytime fatigue and perceptions of pain. Thus, research testing interventions to improve sleep quality are an important inquiry. While many studies acknowledge the significance of mattresses as a key factor influencing sleep quality there is a scarcity of research focused on determining the most effective mattress design for enhancing sleep quality, reducing perceived pain, and improving overall health. This gap is pronounced in studies conducted within natural home settings, involving nonclinical populations, and employing a combination of both validated objective and self‐report assessments, as opposed to controlled environments like hospitals and laboratories. Furthermore, many manufacturers claim health benefits with a particular mattress; however, insufficient research exists to support these claims. Thus, the purpose of this quasi‐experiment was to address these limitations by examining the effectiveness of a pressure‐releasing medium‐firm grid mattress on sleep and health outcomes in adults with nonclinical insomnia in the home environment.

We found that compared to baseline, sleeping on the grid mattress resulted in significant improvements in nighttime sleep quality/quantity and pain, as well as corresponding improvements in daytime mood (i.e., tension, anger, vigor, fatigue, and confusion), perceived stress, daytime fatigue, and pain. Consistent with other research, the mattress is an important environmental component that can improved sleep quality, with mattress firmness being a key aspect.^24^

Of importance, the self‐report sleep data were supported by the objective wearable results from the Oura Ring. In contrast to in‐lab polysomnography studies, in which participants sleep overnight in an unusual sleep environment while being wired to be monitored, the use of a validated multisensory wearable technology enabled tracking of objective sleep in a natural and less invasive way. In‐lab studies offer reliable, robust, and more complete information about the physiological micro‐ and macro‐structure of sleep, but wearables bring unprecedented advantages when studying sleep in ecological environments. Of importance, validation studies of the Oura Ring provide strong support for its accuracy. Specifically, the Oura Ring provided objective data that the participants sleep duration, time awake (i.e., time spent awake in bed before and after falling asleep), light sleep, deep sleep, and total sleep (i.e., amount of time spent in light, REM, and deep sleep) improved while sleeping on the mattress.

Strength of the study include ecological validity of the objective sleep data that supported the self‐report findings, a longitudinal design, validated self‐report measures, and multiple assessment times to track changes and determine time to effect. Although positive effects were continually obtained during the intervention, the present research is limited by the absence of a control group, blinding, and randomization.

Although other individual differences may influence sleep, our moderator analyses revealed no age, gender, and sleep quality effects on the study outcomes. While individual preferences may be relevant, both our objective and subjective sleep outcomes revealed consistent continual improvements over time. Additionally, research has found that mattress preferences may influence sleep quality.^24^ Thus, future studies are encouraged to investigate mattress preferences along with individual differences on their impact on sleep quality and related health outcomes.

The encouraging results obtained from this longitudinal intervention pave the way for randomized controlled trials. In conclusion, optimizing the external environment with a grid mattress is a simple, effective, and low‐burden intervention to improve sleep quality/quantity and related health outcomes in adults with occasional sleeplessness. Further research is needed to determine the effectiveness of this mattress using randomized controlled trials in a variety of populations (e.g., pregnant women, elderly, college students, back pain suffers) and settings (e.g., nursing homes, hospitals, hotels, college dorms).

## AUTHOR CONTRIBUTIONS

**Michael Breus**: Conceptualization, funding acquisition, methodology, project administration, supervision, validation. **Stephanie L. Hooper**: Investigation, methodology, software, formal analysis, project administration, supervision, resources, data curation. **Tarah Lynch**: Project administration, data curation, supervision, resources, formal analysis, methodology. **Martin Barragan**: Methodology, investigation, project administration, resources. **Heather A. Hausenblas**: Conceptualization, investigation, funding acquisition, writing—original draft, methodology, validation, writing—review and editing, formal analysis, project administration, supervision.

## CONFLICT OF INTEREST STATEMENT

Heather A. Hausenblas, Stephanie L. Hooper, Martin Barragan, and Tarah Lynch declare no conflict of interest. Michael Breus served as a former consultant for Purple, LLC.

## ETHICS STATEMENT

We complied with Wiley's Best Practice Guidelines on Publishing Ethics. The work submitted to Health Science Reports was done in accordance to these guidelines and it was performed in an ethical and responsible way, with no research misconduct.

## TRANSPARENCY STATEMENT

The lead author Heather A. Hausenblas affirms that this manuscript is an honest, accurate, and transparent account of the study being reported; that no important aspects of the study have been omitted; and that any discrepancies from the study as planned (and, if relevant, registered) have been explained.

## Data Availability

Data are available upon request from Heather A. Hausenblas.
